# Dynamic modeling of photoacoustic sensor data to classify human blood samples

**DOI:** 10.1007/s11517-023-02939-3

**Published:** 2023-10-25

**Authors:** Argelia Pérez-Pacheco, Roberto G. Ramírez-Chavarría, Rosa M. Quispe-Siccha, Marco P. Colín-García

**Affiliations:** 1 Unidad de Investigación y Desarrollo Tecnológico (UIDT), Hospital General de México “Dr. Eduardo Liceaga”, Dr. Balmis 148, 06720 Cuauhtémoc, Doctores, Ciudad de México México; 2 Instituto de Ingeniería, Universidad Nacional Autónoma de México, Av. Universidad 3000, 04510 Ciudad Universitaria, Coyoacán, Ciudad de México México; 3 Programa de Maestría y Doctorado en Ingeniería, Universidad Nacional Autónoma de México, Av. Universidad 3000, 04510 Ciudad Universitaria, Coyoacán, Ciudad de México México

**Keywords:** Photoacoustics, Data-driven models, Signal processing, Dimensionality reduction

## Abstract

**Abstract:**

The photoacoustic effect is an attractive tool for diagnosis in several biomedical applications. Analyzing photoacoustic signals, however, is challenging to provide qualitative results in an automated way. In this work, we introduce a dynamic modeling scheme of photoacoustic sensor data to classify blood samples according to their physiological status. Thirty-five whole human blood samples were studied with a state-space model estimated by a subspace method. Furthermore, the samples are classified using the model parameters and the linear discriminant analysis algorithm. The classification performance is compared with time- and frequency-domain features and an autoregressive-moving-average model. As a result, the proposed analysis can predict five blood classes: healthy women and men, microcytic and macrocytic anemia, and leukemia. Our findings indicate that the proposed method outperforms conventional signal processing techniques to analyze photoacoustic data for medical diagnosis. Hence, the method is a promising tool in point-of-care devices to detect hematological diseases in clinical scenarios.

**Graphical abstract:**

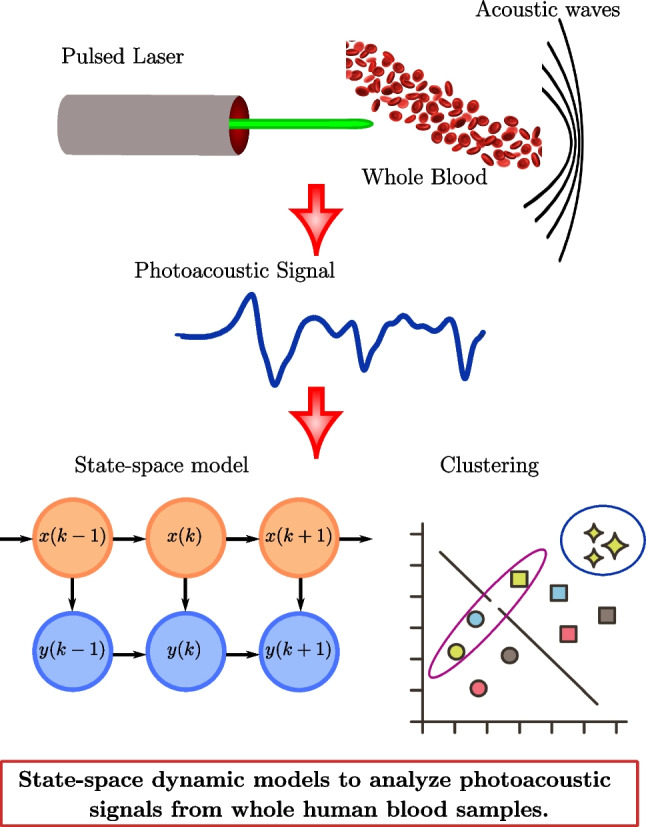

## Introduction

Blood tests are one of the leading medical diagnostic tools and, therefore, one of the foremost clinical practices since it allows monitoring of people’s health, preventing or detecting many diseases or pathologies. Blood is a connective tissue and an extremely dense medium with approximately 45% corpuscular elements and 55% plasma. More than 99% of the corpuscular elements are cells known as erythrocytes or red blood cells (RBC), and less than 1% of the total blood volume corresponds to leukocytes or white blood cells (WBC) and platelets. Usually, the RBC’s diameter is around 7.8 $\mu$m of a healthy individual [1]. The numerical density of human RBC in a healthy adult female is $4.8\times 10^6$ cells/$\mu$L and $5.4\times 10^6$ cells/$\mu$L in a healthy adult male [2], and the mean hemoglobin (Hb) level of women is approximately 12% lower than men [3]. Abnormal levels of some of these elements, due to density, morphology, or size, are indicators of diseases such as anemia, leukemia, heart disease, inflammation, and infections, among others [4].

In the study of blood, different techniques provide valuable information about its composition, function, and diseases [5–7]. For instance, electrical [8], magnetic [9], and optical [10] techniques have been used to obtain several features of the blood. However, these methods are usually expensive and require prior preparation of the samples to be analyzed. Also, some of them have inherent drawbacks, such as low sensitivity and poor specificity [11]. To overcome these issues, current trends employ data analysis to analyze hemodynamic signals for disease prediction [12]. Despite the technological advances related to blood analysis, hematological biometry, the blood smear method, and its visual inspection under the microscope remain the gold standard for evaluating various hematological conditions of blood cells.

PA effect refers to the conversion of light into acoustic waves. These, generated by the optical absorption of pulsed or modulated light, will have the same frequency as the laser’s modulation frequency [13, 14]. The pulsed excitation is widely adopted due to the fact that the signal-to-noise ratio (SNR) of the PA signal is higher than that of the modulated excitation; besides, the typical duration of pulsed laser (10 ns) is lower than both the thermal and stress confinements [15, 16]. In biological tissue, the light is partially absorbed by chromophores, thus converting into heat, resulting in a thermoelastic expansion on the local region. An ultrasound transducer detects these ultrasonic waves for further analysis [17]. The PA technique offers higher spatial resolution and penetration depth than optical techniques, and it is a safe diagnosis tool as it does not use ionizing radiation [18]. In PA sensing, light scattering is discriminated, allowing the acoustic waves to travel long distances without significant distortion or attenuation [19]. Therefore, the pulsed PA technique has been used as a diagnostic tool for different medical applications, including imaging the radial artery, tumors, kidneys, and livers [14, 20].

The PA technique has gained considerable attention for studying blood properties [21], such as observing the cellular components of blood, both in vitro and in vivo [22]. The photoacoustic signal is a product of the incidence of pulsed light that is absorbed by the blood. Therefore, it carries implicit information about its components. The main chromophores are the number of erythrocytes and hemoglobin which are reflected in the features of the PA signal. For example, the PA effect has been employed to analyze erythrocyte osmolarity using microfluidic devices [23], to characterize the viscosity of in vivo blood samples [24], to detect blood glucose [25], and to classify real and fake blood using wavelet neural networks [26]. Nonetheless, many of these studies use animal samples or human blood extracted from a single donor, and the blood is chemically treated to mimic some morphological changes in the RBCs [27–30].

Current biomedical data analysis trends are devoted to developing robust data processing algorithms as novel diagnosis tools. For instance, a multi-modal approach has been introduced to classify images for detecting COVID-19 [31]. Likewise, a deep-learning algorithm has been developed to detect water bodies using remote sensing models [32]. Particularly for PA data, the analyses are divided into time-domain and frequency-domain methods and, more recently, machine learning (ML) models for classification [33, 34]. On the one hand, the temporal profile of the PA signal is analyzed for modeling the signal amplitude and envelope to be related to the size, concentration, and distribution of light absorbers in tissue [35]. Meanwhile, frequency-domain methods are based on analyzing the power spectrum of PA signals to relate their bandwidth with the size of optical absorbers [36]. Lastly, ML has become an attractive alternative to process PA data for robust imaging [37]. Among the available methods, time-domain waveform analysis provides quantitative information about the composition and structure of the tissue under test [38, 39]. However, scarce information is reported about robust methods for PA signal processing in the time domain [40, 41]. Interestingly, as shown in [41], a PA signal can be regarded as the impulse response of a linear model. This approach paves the road for exploiting the dynamics of the PA signals and the flexibility of data-driven methods to discover helpful information in a low-dimensional space [42–44]. Nonetheless, studying human blood samples using the PA effect joint with automated data analysis for diagnosis requires more research attention.

Herein, to the best of our knowledge, we introduce the first results on data-driven methods to analyze PA signals measured from real human blood samples. The proposed method provides a set of parameters related to the physiological conditions of five blood classes, thus serving as features for data classification. In summary, the proposal has the following contributions:

- A subspace method automatically retrieves state-space (SS) models from data, encoding the impulse response features of PA signals, contrary to the conventional methods in PA sensor data analysis.
- The SS model parameters are directly related to the composition and structure of human blood samples by modeling the temporal profile of PA signals.
- The model-based analysis is tested to extract features in five conditions of human blood: (a) female, (b) male, both healthy subjects, and from people with a diagnosis of, (c) microcytic anemia, (d) macrocytic anemia, and (e) leukemia.
- Thus, the SS model-based analysis outperforms the time-domain, frequency-domain, and time-series features when processed using linear discriminant analysis (LDA) for data classification.

The rest of the paper is organized as follows. Section 2 introduces the basis of modeling and the subspace identification algorithm. The experimental details and sample collection are presented in Sect. 3. The experimental findings and a thorough discussion are shown in Sect. 4. Finally, Sect. 5 is devoted to the conclusions.

## Theoretical background

### Photoacoustic effect

The photoacoustic effect establishes that acoustic pressure $p({\vec {r}},t)$ propagates at a position $\vec {r}$ and time *t* according to

1 $$\begin{aligned} \left( \nabla ^2-\frac{1}{v_s^2} \frac{\partial ^2}{\partial t^2}\right) p(\vec {r},t) = -\frac{\beta }{C_p}\frac{\partial ^2H(\vec {r},t)}{\partial t^2}, \end{aligned}$$

where $v_s$ is the speed of sound, $\beta$ denotes the thermal coefficient of volume expansion, ${C_p}$ is the specific heat capacity at constant pressure, and *H* is the heating function. A well-known solution for Eq. 1 involves a delta heating response function $p_\delta (\vec {r},t)$. Hence, the PA signal is computed by the following convolution integral [45]:

2 $$\begin{aligned} p(\vec {r},t)=\int _{-\infty }^{\infty }s(\tau )p_\delta (\vec {r},t-\tau )d\tau , \end{aligned}$$

with $\tau$ a time-dependent integration variable. Experimentally, Eq. 2 is equivalent to the measurement, at a sampling rate $t_s$, of the response of a linear-time-invariant (LTI) system given by

3 $$\begin{aligned} y_{\text {PA}}(k) = u(k)\star h(k)+n(k). \end{aligned}$$

By inspection, it follows that the measured signal $y_{\text {PA}}(k)$ results from the convolution $\star$, of the excitation signal $u(k)=s(\tau )$ with the impulse response function $h(k)=p_\delta (\vec {r},t-\tau )$, and *n*(*k*) is the measurement noise. From Eq. 3, the goal is to find *h*(*t*), such that it fully characterizes the measured PA signals. For this purpose, a SS model has several advantages, such that it could be a flexible approach to describe measurements by capturing the underlying dynamics in a structured form [46].

### State-space model

Given the set of sensor data $\{y_{\text {PA}}(k)\}$ for $k\!=\!(0,1,2,\dots ,N)$, that is, a PA signal measured at a sampling rate $t_s$; then, it can be regarded as the impulse response of a dynamical system with the form

4 $$\begin{aligned} x(k+1)&= Ax(k) + Bu(k),\end{aligned}$$

5 $$\begin{aligned} y(k)&= Cx(k), \end{aligned}$$

which is a SS model. Wherein, $x(k) \in \mathbb {R}^{n\times N}$ are the system states, $y(k) \in \mathbb {R}^{N\times 1}$ is the output sequence to be estimated, and $u(k)\in \mathbb {R}^{N\times 1}$ is the excitation signal. Hence, the dynamic of the PA signal is fully described by the system matrix *A*, the output matrix *C*, and the input matrix *B*. In this way, by estimating the triplet, $\Lambda = \{A,C, B\}$, it is then possible to compute the output sequence *y*(*k*), such that it approximates the measured PA signal, $y(k)\approx y_{\text {PA}}(k)$. This latter is measured directly from blood samples under different physiological conditions.

### Subspace identification algorithm

To estimate the SS model in Eqs. 4 and 5, we use the numerical algorithm for subspace state-space system identification (N4SID) method [47–49] owing its outstanding numerical features. The subspace algorithm works around the measured PA signal $y_{\text {PA}}(k)$ to construct a block Hankel matrix $\mathcal {H}$ defined as

6 $$\begin{aligned} \mathcal {H} = \begin{bmatrix} y_0 &{} y_1 &{} \cdots &{} y_{j-1}\\ \vdots &{} \vdots &{} \ddots &{} \vdots \\ y_{i-1} &{} y_i &{} \cdots &{} y_{i+j-2}\\ \hline y_i &{} y_{i+1} &{} \cdots &{} y_{i+j-1}\\ \vdots &{} \vdots &{} \ddots &{} \vdots \\ y_{2i-1} &{} y_{2i} &{} \cdots &{} y_{2i+j-2}\\ \end{bmatrix} = \begin{bmatrix} Y_p \\ \hline Y_f \end{bmatrix}, \end{aligned}$$

where $Y_p \in \mathbb {R}^{li\times j}$, $Y_f \in \mathbb {R}^{li\times j}$, and the subscripts *p* and *f* denote past and future sequences, respectively, of the PA signal. Also, the input signal and the state sequence are arranged as in Eq. 6. Thereby, one can compute the oblique projection $\mathcal {O}_i$ of Eq. 6 given by

7 $$\begin{aligned} \mathcal {O}_i = Y_f /Y_p, \end{aligned}$$

where $\mathcal {O}_i$ is the projection of the future output space $Y_f$ into the past space $Y_p$. Afterwards, the method performs a singular value decomposition (SVD) of $\mathcal {O}_i$, as follows:

8 $$\begin{aligned} W_1\mathcal {O}_i W_2 = U \Sigma V^\top , \end{aligned}$$

where $W_1$ and $W_2$ are weighting matrices of the singular values, $U\in \mathbb {R}^{m\times n}$ and $V\in \mathbb {R}^{n\times n}$ are orthonormal matrices, and $\Sigma \in \mathbb {R}^{n\times n}$ is the a diagonal matrix of singular values. As the main result, SVD retrieves the number of significant singular values in $\Sigma$, which corresponds to an estimate of the SS model order $\hat{n}$ [50]. Thus, the order represents the number of states describing the PA signal.

**Fig. 1:**
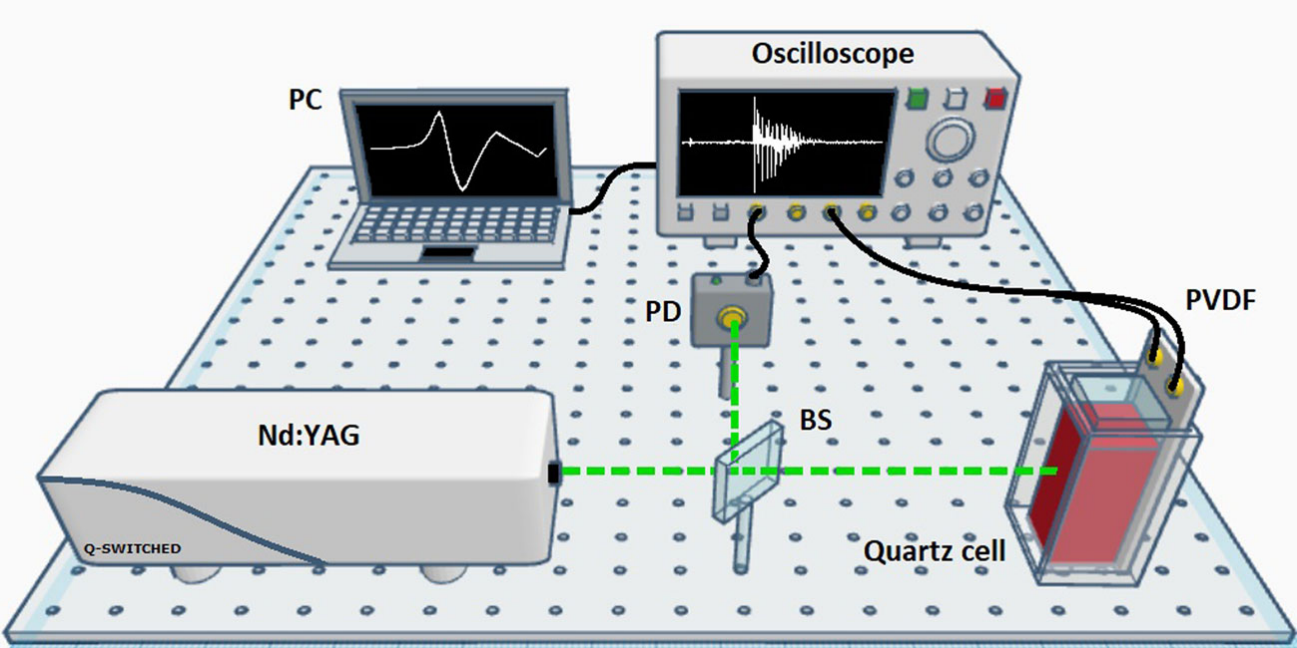
Experimental setup of the PA sensor setup. PC, personal computer; PD, photodiode; BS, beam splitter; PVDF, polyvinylidene fluoride sensor

On the other hand, let be $\Gamma _i\in \mathbb {R}^{li\times n}$ the extended observability matrix defined as

9 $$\begin{aligned} \Gamma _i = \begin{bmatrix} C&CA&CA^2&\cdots&CA^{i-1} \end{bmatrix}^\top . \end{aligned}$$

By construction, the projection $\mathcal {O}_i$ is equal to the product of the observability matrix $\Gamma _i$ and the state sequence $\hat{X}_i=\left( \hat{x}_i \quad \hat{x}_{i+1} \quad \cdots \quad \hat{x}_{i+j-1} \right)$, given by

10 $$\begin{aligned} \mathcal {O}_i = \Gamma _i \hat{X}_i . \end{aligned}$$

Therefore, following the SVD in Eq. 8, the observability matrix can be reconstructed via the singular values of $\mathcal {O}_i$ as follows:

11 $$\begin{aligned} \Gamma _i = W_1^{-1}U\Sigma ^{1/2}. \end{aligned}$$

Also, the projection in (7) is useful to estimate the state sequence since we have that

12 $$\begin{aligned} \hat{X}_i = \Gamma _i^\dagger \mathcal {O}_i, \end{aligned}$$

where the symbol $\dagger$ stands for the pseudo-inverse of $\Gamma _i$. Therefore, for each time instant, the last row of $\Gamma _i$ is removed, thus leading to $\Gamma _{i-1}$. This latter is useful to compute the next state sequence as

13 $$\begin{aligned} \hat{X}_{i+1} = \Gamma _{i-1}^\dagger \mathcal {O}_{i-1}, \end{aligned}$$

where $\mathcal {O}_{i-1}=Y_f^- /Y_p^+$, with $Y_f^-\in \mathbb {R}^{l(i-1)\times j}$ and $Y_p^+\in \mathbb {R}^{l(i+1)\times j}$ are formed by shifting the first block row of $Y_f$ to the last row of $Y_p$ in Eq. 6.

**Table 1:** Hematological biometry data of blood samples labeled in five classes: healthy female (HF), healthy male (HM), microcytic anemia (A1), macrocytic anemia (A2), and leukemia (L)

Parameters	Healthy	Healthy male	Microcytic	Macrocytic	Leukemia
	female (HF)	(HM)	anemia (A1)	anemia (A2)	(L)
	(*N*=10)	(*N*=10)	(*N*=4)	(*N*=4)	(*N*=7)
	M±SD	M±SD	M±SD	M±SD	M±SD
Age (years)	$35\pm 8$	$32\pm 8$	$29\pm 9$	$48\pm 26$	$54\pm 21$
Leukocytes					
($\times 10^3/\mu$L)	$7.3\pm 1.6$	$7.1\pm 1.4$	$5.1\pm 2.1$	$4.3\pm 1.8$	$62.3\pm 59.6$
RI [4.5-11]					
RBCs					
($\times 10^6/\mu$L)	$4.9\pm 0.2$	$5.4\pm 0.2$	$3.4\pm 1.3$	$2.7\pm 0.6$	$2.9\pm 0.8$
RI [4.5$-$5.2]					
Hb (g/dL)					
RI [11$-$18.8]	$15.0\pm 0.9$	$17.1\pm 0.6$	$7.4\pm 1.7$	$8.5\pm 1.6$	$9.4\pm 2.4$
HTC (%)					
RI [35-54]	$44.2\pm 2.6$	$49.7\pm 1.8$	$23.3\pm 6.2$	$26.1\pm 4.2$	$28.4\pm 7.5$
MCV (fL)					
RI [80$-$95.9]	$90.3\pm 3.5$	$91.4\pm 4.7$	$71.7\pm 13$	$99.2\pm 16.5$	$96.8\pm 3.8$

M = mean value, SD = standard deviation

**Note:** RBCs, erythrocytes; Hb, hemoglobin; HTC, hematocrit; MCV, medium corpuscular volume; RI, reference interval

**Fig. 2:**
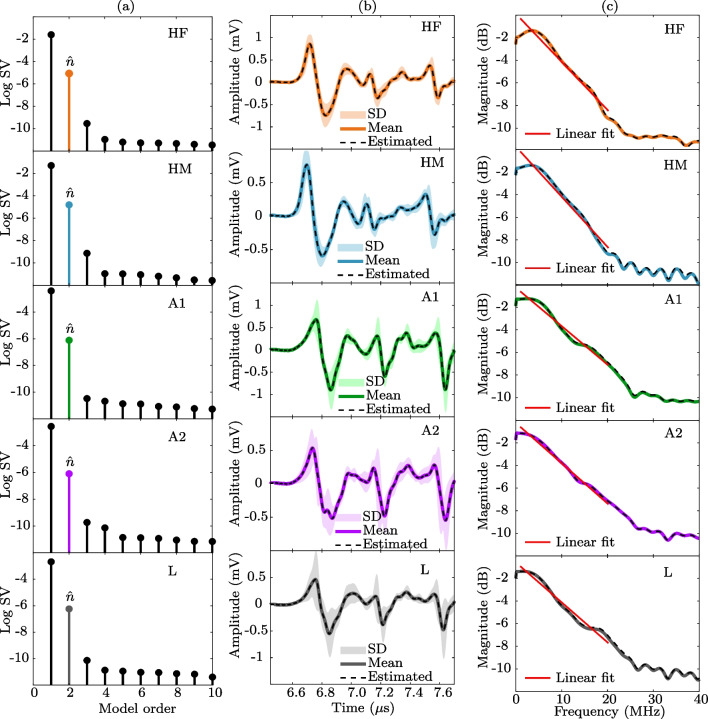
Modeling results of mean PA signals for five blood classes: healthy female (HF), healthy male (HM), microcytic anemia (A1), macrocytic anemia (A2), and leukemia (L). Subplot **a** shows the SVD results indicating an estimated model order $\hat{n}=2$. Subplot **b** depicts the mean value of measurements (solid line), the uncertainty (shaded area), and the mean value of the modeled signal (dashed line). Subplot **c** shows the mean spectrum from measurements (solid line), the mean estimated output spectrum (dashed line), and the spectral linear fitting in the band from 1 to 20 MHz (straight line)

**Fig. 3:**
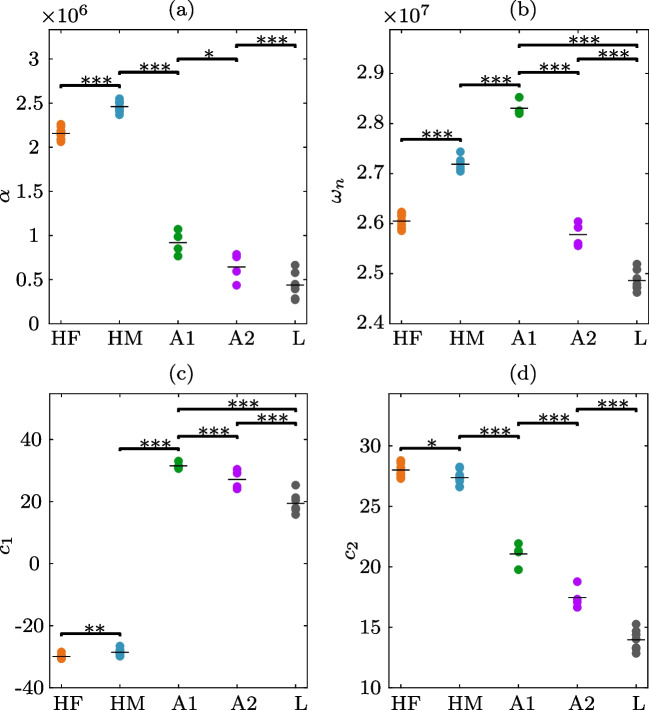
Statistical analysis of the parameters retrieved by the state-space models describing the PA sensor data for five blood classes: healthy female (HF), healthy male (HM), microcytic anemia (A1), macrocytic anemia (A2), and leukemia (L). The estimated values are shown with dots alongside a thin horizontal line indicating the sample mean. For the parameters $$\mathcal {P}\in \mathbb {R}^4$$: **a** decay rate, **b** natural frequency, **c** first mode, and **d** second mode, the statistical significance is explained by $$*\; p< 0.05, \; **\; p<0.01, ~\text {and} \; ***\;p<0.001$$

Once the observability matrix and the state sequence are retrieved, it is then possible to estimate the system matrices $\Lambda =\{A,C, B\}$. For this purpose, we can formulate the following linear problem:

14 $$\begin{aligned} \begin{bmatrix} \hat{X}_{i+1} \\ Y_{i\mid i} \end{bmatrix} = \begin{bmatrix} A &{} B \\ C &{} 0 \end{bmatrix} \begin{bmatrix}\hat{X}_{i}^\dagger \\ U_i\end{bmatrix} + \begin{bmatrix} W \\ V \end{bmatrix}, \end{aligned}$$

where $Y_{i\mid i}$ is a block Hankel matrix with only one row of outputs, *W* is the process noise, and *V* is the measurement disturbance. Furthermore, the problem in Eq. 14 is simplified by neglecting *W* and *V*, as they are considered as zero-mean white noise sequences, uncorrelated with the system states, and do not influence the PA signals owing a high SNR. As a result, the estimated system, input, and output matrices, *A*, *B*, and *C*, are then transformed to its continuous-time representation using the zero-order-hold (ZOH) approximation. This transform allows us to provide a physical model for PA signals, with $A'$, $B'$, and $C'$ the continuous-time-related matrices.

## Materials and methods

### Photoacoustic sensor setup

Figure 1 shows the custom experimental setup to detect PA signals from human blood samples. A Q-switched pulsed Nd:YAG laser at 532 nm was used as the excitation source with a pulse width of 5 ns and a pulse repetition frequency of 10 Hz. The laser fluence was 14.32 mJ/cm$^2$ per pulse, which was measured using a radiometer (UM-B, Gentec-EO). A film transducer of polyvinylidene fluoride (PVDF) (LDT1-028K) with a central frequency of 3.2 MHz was used to capture the PA signal of the blood. In turn, the PVDF film was coupled to one of the faces of a quartz cell (Sigma-Aldrich Z276669) with dimensions of $w=1\times l=1\times h=5$  cm. Each blood sample (3 ml), previously homogenized and without treatment, was carefully deposited inside the quartz cell. The PVDF film transducer was homogeneously coupled to the quartz cell using a double-sided adhesive, and both were held securely within a custom-made PLA container shell. The PA signals were digitized and stored in an oscilloscope (Tektronix DPO 5204B, USA) with 2 GHz bandwidth and a sample rate of 10 GS/s. Throughout the complete set of experiments, the laser beam, energy per pulse, fluency, and power remained constant. Therefore, the PA signal features only depended on the number of absorbers irradiated by the laser beam. Also, the SNR of the PA signals for blood samples was high, so it was not necessary to introduce any amplification stages in our experiment. Finally, the PA signals were acquired by a personal computer (PC) with an Intel™ Core™ i5-7260U CPU at 2.20 GHz, 12 GB in RAM, and an NVIDIA GeForce GTX 1080 GPU. The subspace algorithm and the statistical analysis were implemented in MATLAB™ R2019b.

### Human blood samples

A total of thirty-five whole human blood samples were studied to be analyzed for the first time using the proposed data-driven modeling method. Twenty of these were from healthy donors, ten women and ten men, eight from patients with a diagnosis of anemia, four with microcytosis and four with macrocytosis, and seven from patients with a diagnosis of leukemia. For each one, the correspondent PA signal was obtained in the time domain. The human blood samples were extracted by venipuncture from patients and healthy volunteers and were collected in a Vacutainer™ tube with an anticoagulant (ethylenediaminetetraacetic acid, EDTA). EDTA vacutainer tubes have the authorization of the FDA (Food and Drug Administration) to be used in clinical laboratories since they do not present any risk for the operator and the anticoagulant does not produce alterations in the cells, such as hemolysis, platelet aggregation, or any morphological changes. Hematic biometry was routinely obtained in all samples of this study, and for healthy blood samples, serological studies were performed before acceptance in the Blood Bank. Table 1 summarizes the parameters obtained from hematological biometry of the blood samples used in this work. The samples are labeled according to their physiological status as healthy female (HF), healthy male (HM), microcytic anemia (A1), macrocytic anemia (A2), and leukemia (L).

The healthy blood samples were measured after the serological studies, within 2 h after collection by personnel of the Blood Bank. Meanwhile, the blood samples with a diagnosis of anemia and leukemia were measured 24 h after collection; during that time, they were kept in refrigeration at 4 °C. Before starting with the PA measurements, all blood samples were homogenized, making gentle oscillatory movements of the tube to avoid hemolysis, and then deposited inside the quartz cell. The study protocol was approved by the Ethical, Research, and Bioethics Committees of the Hospital General de México “Dr. Eduardo Liceaga” (No. DI/22/301/03/40). All experiments were performed in accordance with human biomedical research guidelines.

## Results and discussion

### Model estimation of human blood samples

Experimentally, the PA signals were measured using the setup shown in Sect. 3 A, at a sampling rate of $t_s = 0.2$ ns and further segmented into a time window of $\sim 1.2~\mu$s wide (from 6.5 to 7.7 $\mu$s) for each class of human blood. The subspace algorithm then processed the PA signals to retrieve the order and the estimated output of the SS model describing the dynamics. In Fig. 2, we show the results of modeling the measured PA signals using the subspace identification algorithm (see Algorithm 1 in Appendix). On the one hand, Fig. 2a shows the SVD plot of the logarithmic magnitude for ten singular values (SV) representing the order of the SS model in (4) and (5). Interestingly, for all human blood samples, the first two SV are significant since their magnitude is, on average, four orders larger than the other singular values. Thus, the magnitude gap between the singular values 2 and 3 indicates that the estimated SS model has a second-order structure given that $\hat{n}=2$. On the other hand, once the model order was estimated, the subspace algorithm retrieved the SS model output which corresponds to an estimate of the PA signal. Hence, Fig. 2b depicts the mean value of the measurements (solid line) and its uncertainty (shaded area), as well as the mean value of the modeled signals (dashed line) using the subspace algorithm. To further assess the performance of the model-based analysis, we computed the power spectral density (PSD) of the measured and estimated PA signals. The resultant spectra are shown in Fig. 2c for the raw signal (solid line) and the mean magnitude of the output signals from the SS model (dashed line). Also, to obtain quantitative information in the frequency domain, we performed a linear model fitting in the band from 1 to 20 MHz, denoted by the straight line of Fig. 2c.

In the case of healthy people, the PA signals were divided by sex since, according to the literature, the number of RBCs and the Hb level is higher in men than in women. Figure 3b HF and HM show the envelope of the signals of healthy people, which, despite their biological variability, are very similar to each other, independently of sex but slightly different arrival time 6.56 ± 0.01 and 6.59 ± 0.01 $\mu$s for females and males, respectively. For anemic blood samples, the PA signals were divided in microcytic (A1) and macrocytic (A2), according to the average size and volume of RBC’s determined by the parameter of medium corpuscular volume (MCV) whose reference value is between 80 and 95.9 fL and the pathologist’s evaluation by peripheral blood smear. Then, the MCV average was 71.7±13 fL and 99.2±16.5 fL for A1 and A2 samples, respectively. In these cases, Fig. 2b A1 and A2 show how the amplitude of PA signals is less than that of blood from healthy people. Hb levels in the blood with anemia are deficient because the bone marrow does not produce enough RBC to replace those destroyed, thus leading to an alteration of their morphology in some cases. Since Hb is the main absorber of incident light in RBC, its overall reduction, in turn, considerably decreases the amplitude of the PA signal. As shown in Fig. 2b A1 and A2, the mean amplitude of PA signals exhibits more significant variability due to the particles’ shape, size, and distribution. On the other hand, the PA average signals of A2, unlike A1, present a mild oscillation in the first minimum. This situation could indicate that the size of the particles, specifically of the RBCs, modifies the dynamics of the acoustic wave. Therefore, there is a straightforward relationship between the amplitude of the PA signal, the shape, and the size of the RBC [51–53]. This fact is interesting since analyzing the PA signal in the time domain could identify abnormal values associated with diseases such as sickle cell anemia or thalassemia [54, 55].

Figure 2b L shows the mean of PA signals in blood samples from patients diagnosed with leukemia. In this case, it is observed that the PA signal has the highest standard deviation because each sample varies from the other in amplitude, shape, and arrival time. Moreover, the PA signals contain all the information regarding the sample, both for absorbing and non-absorbing chromophores, so the morphology and interaction resulting from the different cells and blood compounds will modify the final shape of the PA signal. Cancer cells also affect the production of RBC and, therefore, the Hb levels. Hence, the mean PA signal for the blood with leukemia is also affected by WBC, which shows a decrease in their amplitude and changes in their oscillations relative to the average blood of healthy samples.

To assess the goodness of the method, we compute the normalized root mean square error (NRMSE) to indicate how well the model response matches the measurement data. The NRMSE is given by

15 $$\begin{aligned} \textrm{NRMSE}=\left( 1-\frac{\Vert y_{\text {PA}}-\hat{y} \Vert }{\Vert y_{\text {PA}} - \text {mean}(y_{\text {PA}})\Vert }\right) \cdot 100\%, \end{aligned}$$

where $y_{\text {PA}}$ and $\hat{y}$ are the measured and model output PA signals, respectively, whereas $\Vert \cdot \Vert$ denote the norm of a vector and $\textrm{mean}(\cdot )$ is the signal average. Thus, the NRMSE quantifies the similarity among signals and allows us to verify the correctness of the retrieved SS model. In Table 2, we summarize the NRMSE for the five blood classes.

**Table 2:** Normalized root mean square error (NRMSE) to indicate the similarity among the modeled and measured PA signals using the subspace algorithm

Blood sample	NRMSE (%)
Healthy female (HF)	99.01
Healthy male (HM)	98.95
Microcytic anemia (A1)	98.12
Macrocytic anemia (A1)	97.65
Leukemia (L)	97.30

### Parametric analysis of state-space models

The estimated SS model by the subspace method has a free-form structure and lacks physical interpretation. However, it is possible to consider that a PA signal behaves as the output of a mechanical system exhibiting an underdamped response. Following this rationale, a similarity transformation, $\mathcal {T}$, can be applied to the estimated SS model such that the system and output matrices become

16 $$\begin{aligned} \mathcal {T}(A')&\rightarrow \bar{A} = \begin{bmatrix} -1/\alpha &{} \omega _n \\ -\omega _n &{} -1/\alpha \end{bmatrix}, \end{aligned}$$

17 $$\begin{aligned} \mathcal {T}(C')&\rightarrow \bar{C} = \left[ c_1 \;\; c_2\right] , \end{aligned}$$

which is now the modal form of the SS representation. Therein, $\alpha$ is the decay rate, and $\omega _n$ is the natural frequency, whereas $c_1$ and $c_2$ are constants relating to how modes are mixed in the PA signal. Under these conditions, the SS model has a twofold action: (i) reduces the dimensionality of a PA signal and (ii) allows for feature extraction with only four parameters $\mathcal {P}=\{\alpha ,\;\omega _n,\; c_1,\; c_2\;\}$. To analyze the difference among PA signals, we considered the set of parameters $\mathcal {P}\in \mathbb {R}^4$ for each blood sample. Differences between groups were analyzed using a 1-way analysis of variance (ANOVA) followed by the Bonferroni post hoc test [32]. The *p*-value of at least $p< 0.05$ was considered significant. Figure 3 shows the scatter plot of the parameters describing the dynamics of the PA signals for the five blood classes: healthy female (HF), healthy male (HM), microcytic anemia (A1), macrocytic anemia (A2), and leukemia (L).

#### Decay rate: $\alpha$

In Fig. 3a, the scatter plot shows the decay rate parameter, which determines how fast the amplitude of the PA signal tends to zero as time grows. It is worth noticing how $\alpha$ can differentiate, with a significance of $p<0.001$, three blood classes: (i) healthy female from male blood, (ii) healthy blood from anemia, and (iii) healthy people and anemia from leukemia. Also, this parameter differentiates with $p<0.05$ between A1 and A2. That is, $\alpha$ reflects the blood composition mainly dictated by the number of erythrocytes or red blood cells (RBC). The $\alpha$ parameter could be related to the ability of blood to lay up energy. In healthy blood, as there is a higher density of erythrocytes concerning blood with anemia, the propagation effect of the acoustic wave is large as can be seen with a shorter arrival time. On the other hand, for blood with leukemia, the number of erythrocytes is even lower than in anemia, which can be interpreted as a decrease in energy storage capacity.

**Fig. 4:**
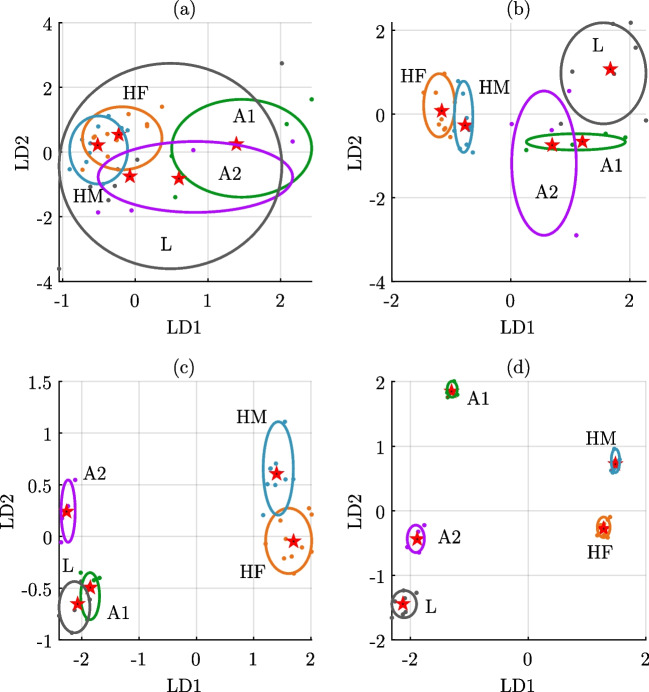
Dimensionality reduction given by two linear discriminants, LD1 and LD2 for five blood classes: healthy female (HF), healthy male (HM), microcytic anemia (A1), macrocytic anemia (A2), and leukemia (L). **a** LDA results for time-domain features. **b** LDA results for frequency-domain features. **c** LDA results for ARMA model-based parameters. **d** LDA results for state-space model-based parameters. The stars are the centroids of each group and the ellipses refer to the 95% confidence

**Fig. 5:**
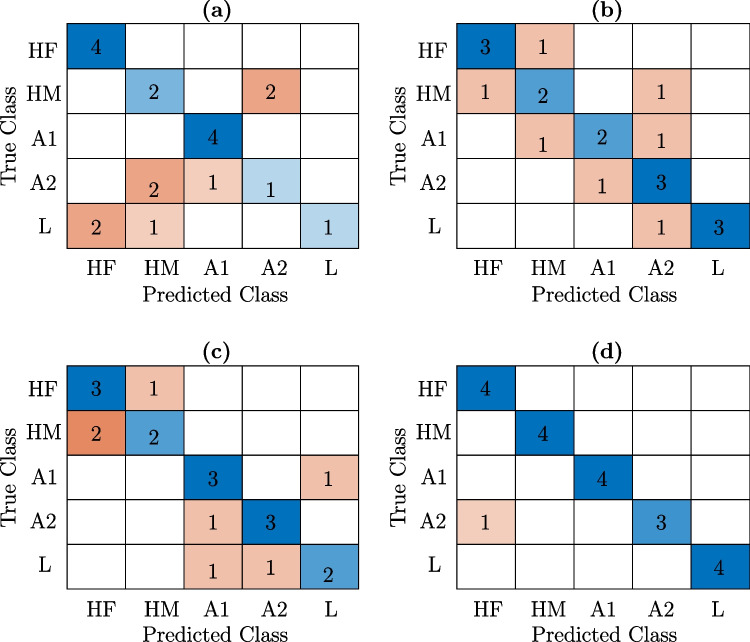
Confusion matrix to test LDA classifier in twenty blood samples: healthy female (HF), healthy male (HM), microcytic anemia (A1), macrocytic anemia (A2), and leukemia (L). **a** LDA test for time-domain features. **b** LDA test for frequency-domain features. **c** LDA test ARMA model parameters. **d** LDA test for state-space model-based features

#### Natural frequency: $\omega _n$

Figure 3b shows the natural frequency behavior for the five blood groups. In this case, $\omega _n$ can be regarded as the frequency, measured in rad/s, at which energy can freely transfer back and forth between the blood sample components. This parameter, in turn, could be associated with the sound propagation speed. As can be seen in Fig. 3b, the parameter $\omega _n$ is in the range of approximately $2.5-3.5\times 10^7$ rad/s for all the blood classes. Moreover, the mean value of the natural frequency allows distinguishing with a significance of $p<0.001$ among healthy blood by gender, among types of anemia, and those from leukemia. This finding follows a naive rationale: the sound speed should be different for blood classes with distinctive composition and structure. Moreover, physically, $\omega _n$ retrieves information on how fast the photoacoustic wave can travel throughout the blood sample.

#### First modal component: $c_1$

The third parameter of the PA signals is related to their structure, given by the output matrix $\bar{C}$ of the SS model. As shown in the scatter plot of Fig. 3c, $c_1$ allows for differentiation among three principal classes. First, the healthy blood of female and male samples have a mean value close to each other, so the difference is explained by a $p<0.01$. Secondly, the anemia condition is substantially different ($p<0.001$) from the healthy samples and can be related to fewer erythrocytes. Finally, leukemia disease shows a decrease in the $c_1$ value, which allows one to differentiate it from the healthy and anemia classes, with a *p*-value of 0.001. Moreover, the two types of anemia are also statistically different among them with $p<0.001$. Interestingly, the parameter $c_1$ retrieves information on the structure of the PA signal, and it could be related to the light absorption in the blood.

#### Second modal component: $c_2$

The next element of the output matrix $\bar{C}$ is the so-called second modal component. Figure 3d depicts the performance of $c_1$ as a function of the blood class. This parameter shows an impressive performance because its value decreases as a function of the physiological condition of the sample. That is, for healthy blood, $c_2$ is larger than an anemia blood sample, and this latter, in average, is also larger than for leukemia. As expected, abnormal blood conditions exhibit a lower concentration of erythrocytes, and hence, $c_2$ can be a parameter modeling such effect. The greater the number of RBC, the larger the value of the second modal parameter. For $c_2$, the statistical difference is moderate ($p<0.05$) between female and male samples and high ($p<0.001$) to distinguish from healthy, anemia, and leukemia blood.

### Dimensionality reduction for human blood classification

One aim of the model-based analysis is to serve as a feature extraction technique for further data classification (see Algorithm 1 in Appendix). Typically, the PA signal analysis is carried out in the time domain (TD) and frequency domain (FD). Hence, based on previous reports [14, 56], we selected some useful features to compare it with the SS model parameters. For TD, the following features were computed straightforwardly from the measured PA signals (see Fig. 2b): (i) maximum amplitude, (ii) time of arrival, (iii) root-mean-square value, and (iv) area under the curve. On the other hand, the FD features were computed from the PSD of the signals (see Fig. 2c), thus leading to (i) spectral slope, (ii) magnitude intercept, (iii) midband fit, and (iv) peak frequency. Also, the PA signals were fitted to an autoregressive-moving-average (ARMA) model with four parameters. The choice of ARMA models is due to their ability to capture the dynamics of the signal, similar to the SS model. Regarding our method, it is worth noticing that the features are those directly retrieved by the SS models and described in Sect. 4.2. Subsequently, the features from TD, FD, ARMA, and SS model-based were fed into linear discriminant analysis (LDA) algorithm for dimensionality reduction and data classification. For this purpose, the hypothesis was that the number of features could be reduced to represent the PA signals in a shallow dimensional space for classification. Figure 4 shows the results retrieved by LDA represented using two discriminants, LD1 and LD2. Therein, the dots represent the measured blood samples for each class, the stars stand for the centroid of each group, and the ellipses refer to the 95% confidence interval for the groups of data.

Firstly, Fig. 4a shows the LDA representation of the TD features. In this case, LD1 and LD2 scores are very close to each other for all classes. Hence, the classifier could not well differentiate among blood samples. This could be attributed due to the variability of the PA-measured signals, which in time domain is more visible than in other domains. Likewise, Fig. 4b depicts the scatter plot for the results retrieved by LDA using the frequency-domain features. In such a case, the ellipses slightly overlap among healthy classes HF and HM, and for anemia samples A1 and A2, thus indicating that all blood classes cannot be correctly discriminated. Nonetheless, it is interesting to observe that using FD features, LDA at least could identify two blood classes, healthy (HF and HM) and diseased (A1, A2, and L). On the other hand, the classification results using ARMA model parameters are shown in Fig. 4c. Therein, it is more evident to see that this dynamical model was able to separate healthy from diseased samples. However, within these two groups, the ARMA parameters are still unable to specifically classify the samples according to the clinical labels. Finally, the SS model-based results are shown in Fig. 4c. From there, it is worth noticing how LDA applied to the SS model parameters can differentiate the five blood classes by well-defined ellipses classifying each group. Furthermore, the variability in the LDA scores is such that the confidence ellipses do not overlap each other. Interestingly, with the proposed model-based approach, one can see that the algorithm can distinguish between two classes of healthy blood (HF and HM) and, also, among three classes of disease (A1, A2, and L). In the first case, the SS model approach agrees with the literature where the amount of hemoglobin in blood is higher in men than in women. Thus, this situation is reflected in the photoacoustic signals and captured by the model parameters with enough sensitivity.

**Table 3:** Comparison of current technologies for blood analysis in disease detection

Technology	Target/disease	Adavantages	Disadvantages
	Anemia	- Multi-desease	
Photoacoustics	and	- Automatic analysis	- Preliminary results
(this work)	leukemia	- Whole blood analysis	- Experimental setup
Optical	RBC counting	- Low-cost	
microscopy	and	- Classification	-Preliminary results
Pellegrino et al. [57]	morphology	- Cell morphology	- Large dataset
Centrifugal		- Whole blood analysis	
microfluidics	Hemoglobin	- Microvolume	- High fabrication cost
Mahmodi Arjmand et al. [58]	quantification	- High sensitivity	- Sample pre-treatment
		- Blood rheology	
Electrical sensor	RBC for hypoxia	- Point-of-care devices	- Bench-top equipment
Liu et al. [59]	in sickle cell	- Label-free	- Sample pre-treatment
	Hemoglobin	- Quantitative results	
Optical sensor	and	- Microvolume	- Preliminary results
Bahadoran et al. [60]	anemia	- High precision	- Artificial samples

According to our findings for human blood classification, the differences in PA sensor data could be related with the blood composition and structure. This situation could be confirmed owing the statistical difference in the SS model parameters (see Fig. 3). More specifically, the PA signals and the model parameters are mainly related to the number of RBC and WBC, Hb level, and size of the erythrocytes. Hence, by carefully analyzing the profile of the PA signal, it retrieves information on the size of RBC, and therefore, the concentration of Hb within them can be detected [55].

To have a more rigorous study, a set of twenty samples was considered to test the performance of the LDA classifier. For this purpose, the trained model in Fig. 4 was used to predict the class of human blood samples. Figure 5 shows the confusion matrices for (a) time-domain, (b) frequency-domain, (c) ARMA model, and (d) SS model features. From these results, it was possible to quantify the classification task through its accuracy, defined as the probability that the model prediction is correct. Thus, the computed accuracy for each kind of features was 60% for time domain, 65% for frequency domain and ARMA, and 95% for the state-space models. Interestingly, it is possible to highlight that a simple classifier managed the PA sensor data with acceptable accuracy. However, one should keep in mind that the feature extraction based on SS models is a crucial step before the classification. Although the number of samples was relatively small due to availability, this work represents a first pilot study with actual human blood. Hence, our findings are promissory toward novel methods to analyze PA data from blood and classify it according to different pathological conditions. That is, we are not dealing with a standardized protocol for studying blood samples, and instead, the data-driven method pretends to be the basis of further and extensive studies.

Finally, we compare our proposal with four technological developments for blood analysis. Among the wide variety of available technologies, we selected those with similar capabilities to our photoacoustic approach. Hence, the revised literature considers blood analysis to assess the composition and structure of blood to detect hematological diseases. For this comparison, we appeal to three qualitative scores highlighting the target of the method/device and their advantages and disadvantages in blood study. It is worth noticing that the selected works show preliminary studies instead of well-known protocols to perform a deep clinical study. Table 3 summarizes the main features of our photoacoustic technology and its comparison with optical microscopy, microfluidics, and electrical and optical sensors.

From Table 3, it is possible to deduce that our proposal outperforms other technologies by providing a multi-disease classification using whole blood without pre-treatment. This goal is achieved by taking advantage of the versatility of PA effect coupled with advanced methods for data modeling and processing.

### Perspectives and challenges in photoacoustic data modeling

The proposed dynamical modeling approach has several advantages compared to the classical methods for analyzing PA data. However, there are some perspectives and challenges that must be taken into account in further works. The data-driven approach in PA could be applied in several clinical and biomedical scenarios, such as medical diagnosis, health monitoring, blood medical research, and development of point-of-care devices. Within this framework, PA analysis would be able to identify specific biomarkers for disease detection and monitoring in the kidney, heart, liver, and blood, for instance. Moreover, the development of the dynamical modeling approach could be integrated into portable devices or handheld non-invasive scanners, allowing a quickly assessment of a patient’s health status or screening for specific conditions. Also, the model-based approach could be an attractive method for PA signal deconvolution, an open problem in PA tomography [61]. Altogether, these improvements could be a significant progress toward novel algorithms for automated analysis in biomedical diagnosis.

Particularly, despite its promising applications, the use of data-driven models for blood sample classification also presents some limitations and challenges. For instance, sample variability requires attention to detect and discriminate outliers to provide accurate results. The data availability can be limited, challenging, and time-consuming, but are necessary to train algorithms with high specificity and accuracy. Also, validating the dynamical models using real-world samples and clinical trials is crucial to ensure their effectiveness in practical settings. Finally, it is worth saying that data-driven methods do not replace medical oversight and expertise but pretend to be complementary tools to support medical decision-making, and the results should always be validated and reviewed by trained healthcare professionals. In summary, data-driven dynamical models have the ability to classify blood samples in several real-world applications, but also present challenges and limitations that must be addressed to ensure their accuracy, validity, and ethics in practical settings.

## Conclusion

In this work, we introduced an attractive method to derive dynamic models of photoacoustic signals to classify human blood samples. We showed how state-space models, estimated via subspace identification, are an attractive method to analyze whole human blood samples using raw photoacoustic data. Also, a parametric analysis was carried out to relate the erythrocytes’ size, shape, and volume in the evaluated blood samples. Moreover, the model parameters served as features of the photoacoustic signals to classify it according to blood physiological conditions. The performance of the model-based approach exhibited superiority when compared with classical time- and frequency-domain features and autoregressive models using linear discriminant analysis. The versatility of our proposal shows its potential to translate into clinical applications for detecting, at the point-of-care, the progress of hematological diseases and tracking the efficacy of treatments. Finally, this work could strengthen novel trends in model-based photoacoustic analysis to speed up the experimental process as a complementary tool for clinical diagnosis.

## Figures and Tables

**Algorithm 1:**
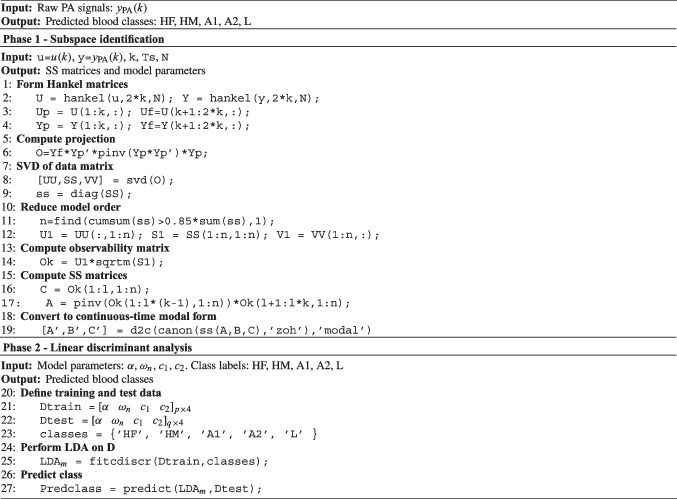
Dynamical model for blood classification.

## Appendix: Dynamical model-based algorithm for blood classification

Once the PA signals are acquired from blood samples, they are the input of the proposed algorithm working in a two-phase procedure as shown in Algorithm 1. Firstly, the raw PA data are fitted to a SS model using the subspace identification method. Thus, the model parameters serve as features describing each blood sample. Afterwards, the features are fed into linear discriminant analysis (LDA) algorithm, which aims to find a linear combination of the features that characterizes the five blood classes, healthy female (HF), healthy male (HM), microcytic anemia (A1), macrocytic anemia (A2), and leukemia (L).
